# Enhanced Nrf2 up‐regulation by extracellular basic pH in a human skin equivalent system

**DOI:** 10.1111/jcmm.16472

**Published:** 2021-03-16

**Authors:** Gunhyuk Park, Byeong Cheol Moon, Dal‐Seok Oh, Yong‐Ung Kim, Moon‐Ki Park

**Affiliations:** ^1^ Herbal Medicine Resources Research Center Korea Institute of Oriental Medicine Naju‐si Korea; ^2^ The Herbal Medicine Research Division Korea Institute of Oriental Medicine Daejeon Korea; ^3^ Department of Pharmaceutical Engineering College of Biomedical Science Daegu Haany University Gyeongsan Korea

**Keywords:** 3D human skin equivalent system, keratinocyte growth factor, nuclear factor erythroid 2‐related factor 2, pH

## Abstract

Extracellular basic pH regulates cellular processes in wounds, and consequently influenced wound healing. Oxidative defence system modulation in the skin helps heal wounds, inhibits skin ageing and improves the skin condition. Moreover, the role of keratinocyte growth factor (KGF) and nuclear factor erythroid 2‐related factor 2 (Nrf2) in antioxidant systems has been reported in various skin models. However, the effects of extracellular basic pH on wound‐ or skin ageing‐related skin damage have not been examined. Thus, we investigated the antioxidant systems affected by extracellular basic pH in a 3D human skin equivalent system (3HSE). Extracellular basic pH decreased KGF expression and enhanced the oxidative defence system, and thus activated Nrf2 in the 3HSE. Additionally, extracellular basic pH and KGF treatment up‐regulated Nrf2 activation and its regulation of the oxidative defence system in the 3HSE. This indicates that Nrf2 up‐regulation is enhanced by reactive oxygen species production, rather than KGF, and by extracellular basic pH of the skin. The inhibition of skin damage through pH imbalance and KGF regulation suggests that the development of pH‐regulating or pH‐maintaining materials may provide effective therapeutic strategies for maintaining a healthy skin.

## 1 INTRODUCTION

Extracellular basic pH plays a significant role in directly and indirectly regulating cellular processes in wounds, and consequently affects the wound‐healing process.^1^ Chronic wounds are ideal microenvironments for bacterial growth.^2^ In fact, bacterial infection is one of the most prevalent causes of poor wound healing.^2^, ^3^ Bacteria in the wound bed can spread to the surrounding tissues, resulting in a localized infection.^3^, ^4^ If this infection is left untreated, the spread of bacteria can result in systemic infection.^4^ It has been reported that basic pH in the wound bed can create an unsuitable environment for wound healing by promoting the growth of pathogenic bacteria.^4^, ^5^ We previously demonstrated that extracellular pH imbalance in the skin can interrupt tissue remodelling by accelerating collagen breakdown.^6^ In an in vitro study and a 3D human skin equivalent system (3HSE), basic pH conditions reportedly increased intracellular reactive oxygen species (ROS) generation and mitogen‐activated protein kinase signalling, whereas weakly acidic pH conditions slightly increased intracellular ROS generation and p38 kinase signalling, but not extracellular signal‐regulated kinases (ERK) and c‐Jun N‐terminal kinase signalling (JNK).^6^ Moreover, in an in vitro study and a 3HSE system, basic pH conditions increased early‐stage apoptosis through apoptosis antigen 1/apoptosis antigen 1 ligand via the modulation of heat‐shock protein (HSP)‐27, HSP‐60 and HSP‐70, which are an important effector of wound healing.^7^ The inhibition of wound healing through pH imbalance and the effects of pH imbalance on the apoptotic processes involved in skin diseases such as atopic dermatitis suggest that the development of pH‐regulating or pH‐maintaining materials may provide effective therapeutic strategies for maintaining a healthy skin.

A recent study revealed a potential cytoprotective effect of keratinocyte growth factor (KGF) on different types of epithelial cells.^8^ KGF is a member of the fibroblast growth factor (FGF) family of mitogens.^8^ While most FGFs influence the proliferation and/or differentiation of various cell types, KGF seems to specifically act on epithelial cells by binding to its high‐affinity receptor, a splice variant of FGF receptor 2.^8^, ^9^ KGF stimulates the proliferation and migration of these cells; however, it also affects the differentiation of cells.^10^ Moreover, it promotes the survival of cells under stress conditions.^11^ KGF expression is up‐regulated in injured and inflamed tissues including the wound‐healing system in the skin.^12^ Moreover, with skin injury, the inhibition of KGF receptor signalling reduced the proliferation of epidermal keratinocytes at the wound edge, substantially delaying wound re‐epithelialization.^12^ Interestingly, in their effort to identify KGF‐regulated genes in keratinocytes, Braun et al^11^ identified the gene encoding the transcription factor NF‐E2–related factor 2 (Nrf2) and determined the related expression of genes encoding ROS‐detoxifying enzymes. Nrf2 plays a key role in the cellular stress response 13. It might be important for wound healing, whereby large amounts of ROS are produced to defend against invading bacteria.^13^, ^14^ Moreover, various key factors involved in wound healing were significantly decreased in early‐stage wounds in Nrf2 knockout mice, whereas the later stages were characterized by prolonged inflammation.^11^, ^15^ Thus, these studies have demonstrated the importance of Nrf2/KGF modulation in maintaining a healthy skin. Despite these possibilities, the association between Nrf2/KGF and pH control has not been studied. Therefore, we examined the influence of the antioxidative defence system by regulating KGF/Nrf2 induced by extracellular basic pH in the 3HSE.

## 2 MATERIALS AND METHODS

### 2.1 Chemical

Dulbecco's modified Eagle medium (DMEM), penicillin‐streptomycin, phosphate‐buffered saline (PBS) and foetal bovine serum (FBS) were purchased from Gibco (MD, USA). Neoderm®‐ED and its regulating media were purchased from TEGO Science (Seoul, South Korea). Hydrochloric acid, MTT, sodium hydroxide, recombinant human KGF (KGF inducer) and dimethyl sulphoxide (DMSO) were purchased from Sigma‐Aldrich (St. Louis, USA). Rabbit anti‐heme oxygenase‐1 (HO‐1), connective tissue growth factor (CTGF), and NAD(P)H:Quinone oxidoreductase‐1 (NQO1) and mouse keratin 14 (K14) antibodies were purchased from Abcam (Cambridge, UK). superoxide dismutase (SOD) and catalase (CAT) activity assay ELISA kit was purchased from BioVision (Milpitas, CA, USA). Biotinylated goat anti‐rabbit antibody, bovine serum albumin (BSA), normal goat serum and VECTASTAIN Elite ABC Kit were purchased from Vector Labs (CA, USA). The Trans‐AM assay Kit for Nrf2 was purchased from Active Motif (Carlsbad, CA). All other reagents used were of guaranteed or analytical grade.

### 2.2 Cell culture and 3D human skin equivalent system

Using Neoderm®‐ED, we generated the 3HSE system. The 3HSE and cell culture systems were established as described previously.^6^, ^7^ Briefly, human primary dermal fibroblasts were cultured in collagen matrix for 1 day. Keratinocytes were then seeded on top of the collagen matrix and co‐cultured for 4 days. Subsequently, human primary epidermal keratinocyte and human primary dermal fibroblast blocks were lifted and exposed to air. The ratio of fibroblast marker to keratinocyte marker was 40:60 (Figure S1). We switched to a new medium without sodium bicarbonate for pH control. The skin equivalent system was then treated at a pH of 6.40‐7.70 using HCl or NaOH and with KGF inducer (25 μmol/L or 50 μmol/L) for 10 days, and the medium was changed every day for 10 days. During these 10 days, the pH level was maintained at approximately ±0.5 degree, and not beyond that. The skin equivalent system was incubated at 37°C and 5% CO_2_. The human skin keratinocyte HaCaT cell line was obtained from CLS Cell Lines Service GmbH (Baden‐Württemberg, Germany) and maintained in DMEM supplemented with 10% heat‐inactivated FBS and 1% penicillin‐streptomycin in 95% air and 5% CO_2_ at 37°C. The cells were treated with KGF inducer for 1 hour; then, they were stimulated at pH 7.90 for an additional 23 hours.

### 2.3 Skin immunohistochemistry analysis

Skin sections were prepared as previously described.^7^ Briefly, 5‐μm‐thick sections of 10% neutral formalin‐fixed, paraffin‐embedded tissues were cut on silane‐coated glass slides, de‐paraffinized three times with xylene and then dehydrated through a graded alcohol bath. Subsequently, the sections were washed with PBS before immunostaining and pretreated by blocking with 1% BSA for 30 minutes to prevent nonspecific binding of the antibodies. The sections were then incubated overnight with the primary anti‐antibody (1:500 dilution) in PBS containing 0.3% Triton X‐100 and normal goat serum, and subsequently with the secondary antibody (1:100 dilution) for 60 minutes, followed by incubation in ABC solution for 1 hour at room temperature. Colour development was performed by incubating the sections with streptavidin for 40 minutes. The slides were incubated for 10 minutes with 50 μg/mL 4′,6‐diamidino‐2‐phenylindole (DAPI). Images were viewed using a microscope (Olympus Microscope System BX53; Olympus, Tokyo, Japan). The results were quantified by measuring the fluorescent density at 40× magnification using ImageJ software (Bethesda, MD, USA); data are presented as a percentage of the pH 7.40 group values.

### 2.4 Detecting KGF, HO‐1 and NQO1 by Western Blotting

Western blotting was performed according to previously published methods.^16^, ^17^, ^18^ The cells and tissues were lysed with protein extraction buffer to obtain whole‐protein extracts. The lysates were separated by 12% SDS‐PAGE and then transferred onto membranes. The membranes were incubated with 3% BSA in TBST for 1 hour. They were then incubated with the primary antibody (1:1000 dilution) overnight at 4°C and then with the HRP‐conjugated secondary antibody for 1 hour. Immunoreactive bands were detected using an ECL detection kit, and the LAS‐4000 mini system (Fujifilm Corporation, Tokyo, Japan) was used for visualization. The intensity of the bands was normalized with that of β‐actin and analysed using Multi‐Gauge software (Fujifilm Corporation, Tokyo, Japan).

### 2.5 Measuring Nrf2, SOD, CAT and GSH levels by ELISA kits

The DNA‐binding activity of Nrf2 was evaluated using a commercially available Trans‐AM Nrf2 kit.^19^, ^20^ Briefly, 15 μg of nuclear extract was incubated with immobilized wild‐type or mutated competitor oligonucleotides with the antioxidant response element (ARE) consensus sequence. The bound Nrf2 was detected using an anti‐Nrf2 primary antibody (1:1000 dilution) and HRP‐conjugated secondary antibody (1:1000 dilution) before chromogenic reaction with TMB substrate, and the absorbance of the solution was measured at 450 nm using a plate reader. Moreover, SOD, CAT and GSH levels in the skin were assessed using a commercially available ELISA assay or array kit, according to the manufacturer's protocol.

## 3 RESULTS

### 3.1 Effect of basic pH on the Nrf2 activity and its regulation of antioxidative response enzyme in the 3HSE

**FIGURE 1:**
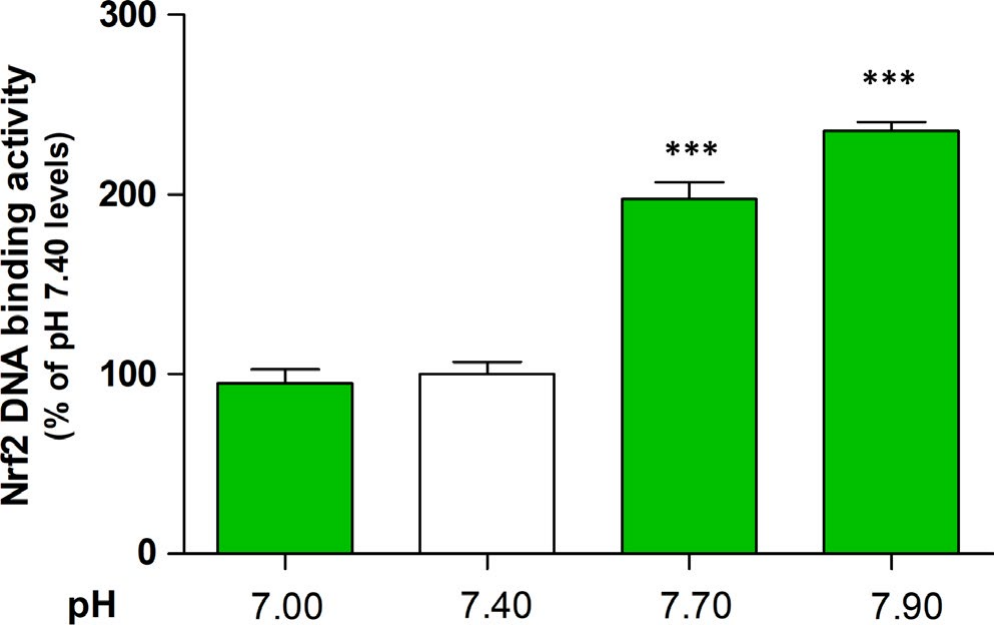
Effects of basic pH on the Nrf2 activity in the 3HSE. Nrf2 DNA‐binding activity was measured using an ELISA kit. The values are presented as mean ± SEM. ***P < .001; one‐way ANOVA, followed by Tukey's post hoc test, was performed using GraphPad Prism software

**FIGURE 2:**
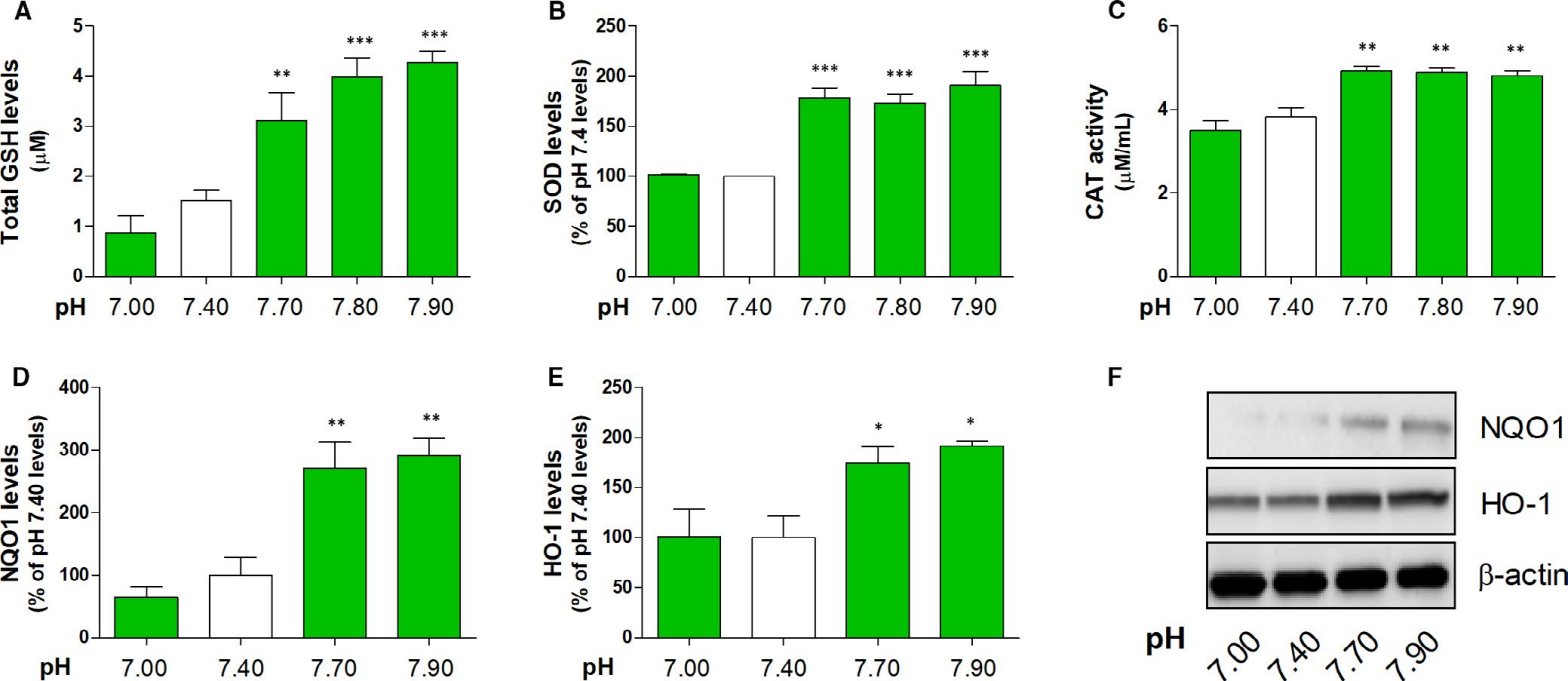
Effects of basic pH on antioxidative defence enzyme levels in the 3HSE. The levels of GSH, SOD and CAT were measured using ELISA kits (A, B and C). The NQO1 and HO‐1 expression levels were measured by Western blotting (D, E and F). The values are presented as mean ± SEM. *P < .05, **P < .01 and ***P < .001; one‐way ANOVA, followed by Tukey's post hoc test, was performed using GraphPad Prism software

We investigated the effect of acidic or basic pH on antioxidative response enzyme and Nrf2 activity by determining the levels of SOD, CAT, GSH, HO‐1, and NQO1 and the DNA‐binding activity of Nrf2 in the 3HSE. The DNA‐binding activity of Nrf2 increased under basic pH conditions in the nuclear fractions (Figure 1 and Table S1). Moreover, the levels of antioxidative response enzymes regulated by Nrf2, namely GSH (Figure 2A and Table S2), SOD (Figure 2B and Table S2), CAT (Figure 2C and Table S2), NQO1 (Figure 2D,F and Table S2) and HO‐1 (Figure 2E,F and Table S2), increased under basic pH conditions compared with those under pH 7.4.

### 3.2 Effect of basic pH on KGF expression in the 3HSE

**FIGURE 3:**
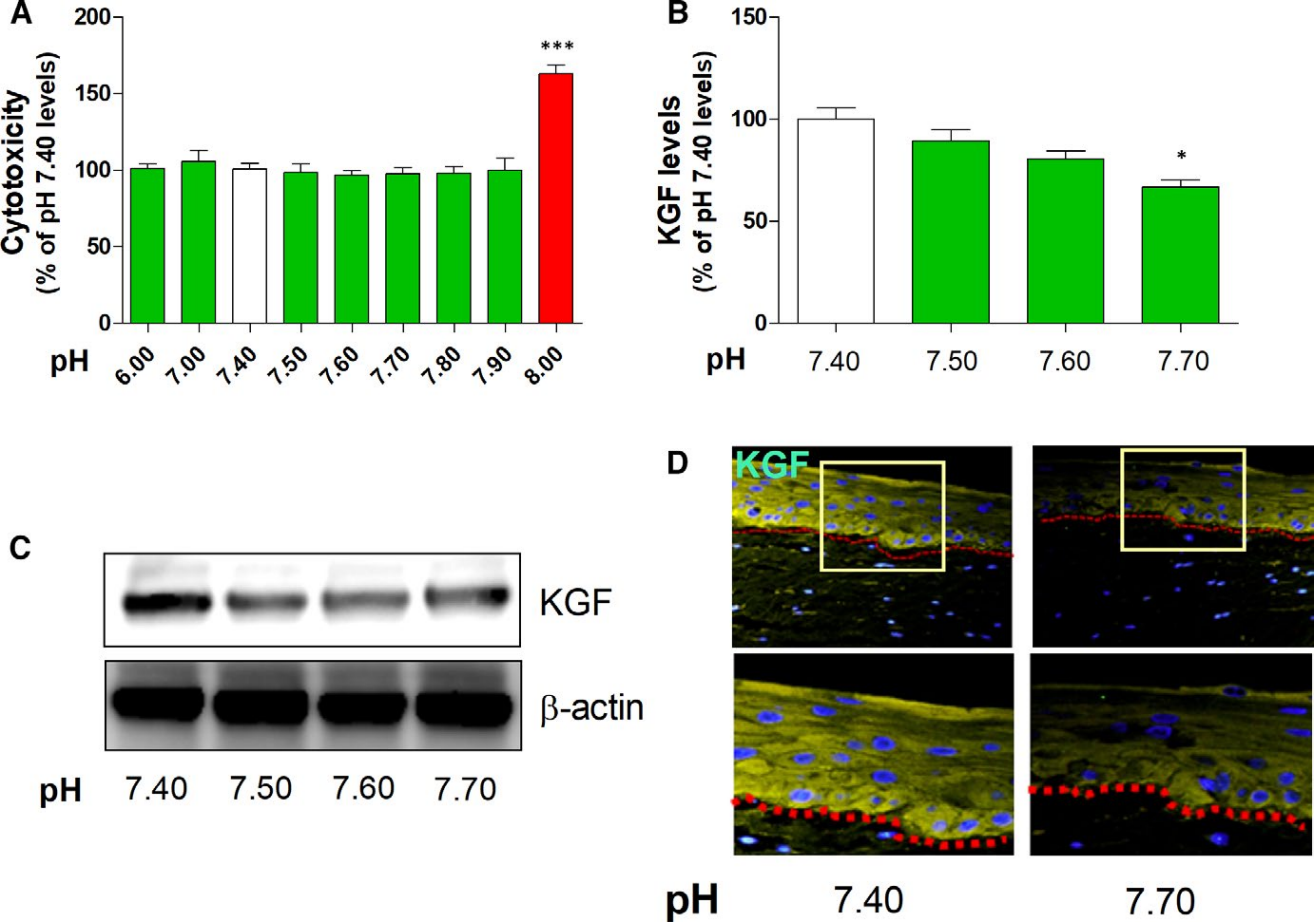
Effects of basic pH on cytotoxicity in the 3HSE. Cytotoxicity under acidic and basic conditions was assessed using the LDH assay (A). The level of KGF expression was measured by Western blotting (B and C). Histological analysis of representative KGF proteins in a section of 3D human skin block treated with basic pH (D). Scale bar = 100 μm. The values are presented as mean ± SEM. *P < .05, **P < .01 and ***P < .001; one‐way ANOVA, followed by Tukey's post hoc test, was performed using GraphPad Prism software

We investigated the effect of acidic or basic pH on KGF expression by determining the levels of KGF using ELISA, Western blotting, PCR and immunocytochemistry. The LDH assay results demonstrated that the viability of the 3HSE was not affected by 24 hours of treatment at pH 6.00‐7.90. However, at pH ~ 8.00, cytotoxicity increased (Figure 3A and Table S3). Moreover, basic pH decreased the KGF level measured using ELISA (Figure 3B and Figure S2) and Western blotting (Figure 3B,C and Table S3), compared with the level measured using other methods at pH 7.4. Furthermore, decreased KGF intensity was observed by immunohistochemistry in the 3HSE (Figure 3D).

### 3.3 Effect of basic pH and KGF inducer on Nrf2 activity and its regulation of antioxidative response enzyme in the 3HSE

**FIGURE 4:**
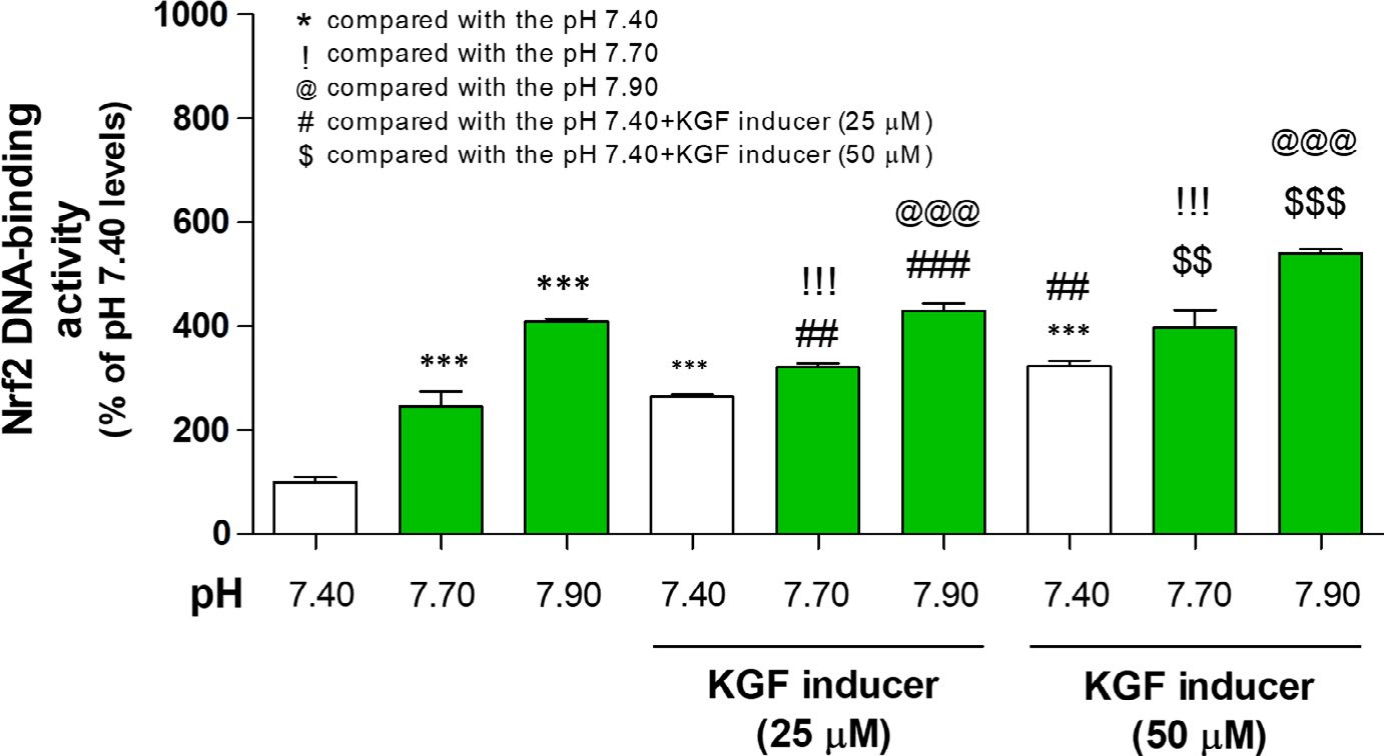
Effects of basic pH and KGF inducer on the Nrf2 activity in the 3HSE. The Nrf2 DNA‐binding activity was measured using ELISA. The values are presented as mean ± SEM. ## or $$*P* < .01 and ***, ###, $$$, &&&, !!! and @@@*P* < .001; one‐way ANOVA, followed by Tukey's post hoc test, was performed using GraphPad Prism software

**FIGURE 5:**
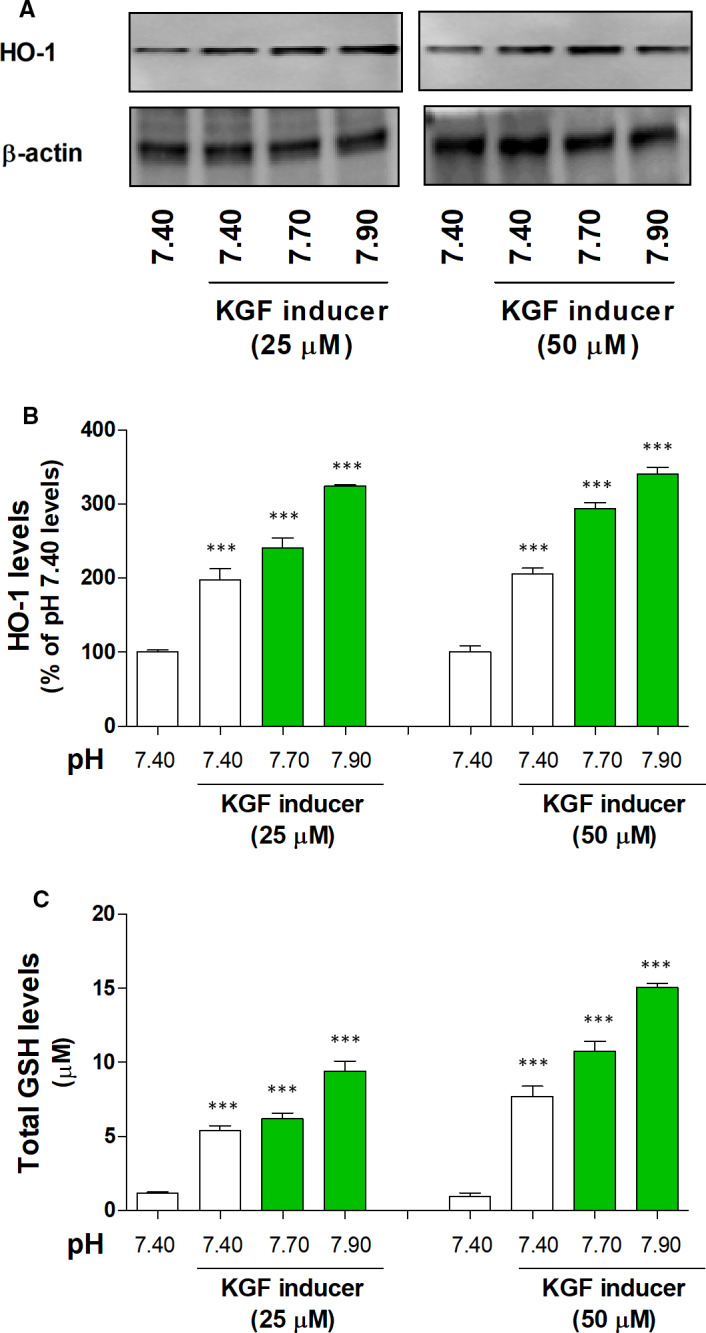
Effects of basic pH and KGF inducer on the antioxidative defence enzyme levels in the 3HSE. The level of HO‐1 expression was measured by Western blotting (A and B). The level of GSH was measured using ELISA kit (C). The values are presented as mean ± SEM. ***P < .001; one‐way ANOVA, followed by Tukey's post hoc test, was performed using GraphPad Prism software

To confirm the effect of basic pH and KGF inducer on the antioxidative response system, we determined the DNA‐binding activity of Nrf2 and the levels of GSH and HO‐1 in the 3HSE. The DNA‐binding activity of Nrf2 increased under basic pH and treatment with 25 and 50 μmol/L KGF inducer (Figure 4 and Table S4). Moreover, the HO‐1 and GSH levels increased under basic pH and treatment with 25 and 50 μmol/L KGF inducer (Figure 5 and Table S5).

### 3.4 Effect of basic pH and KGF inducer on skin proliferation

**FIGURE 6:**
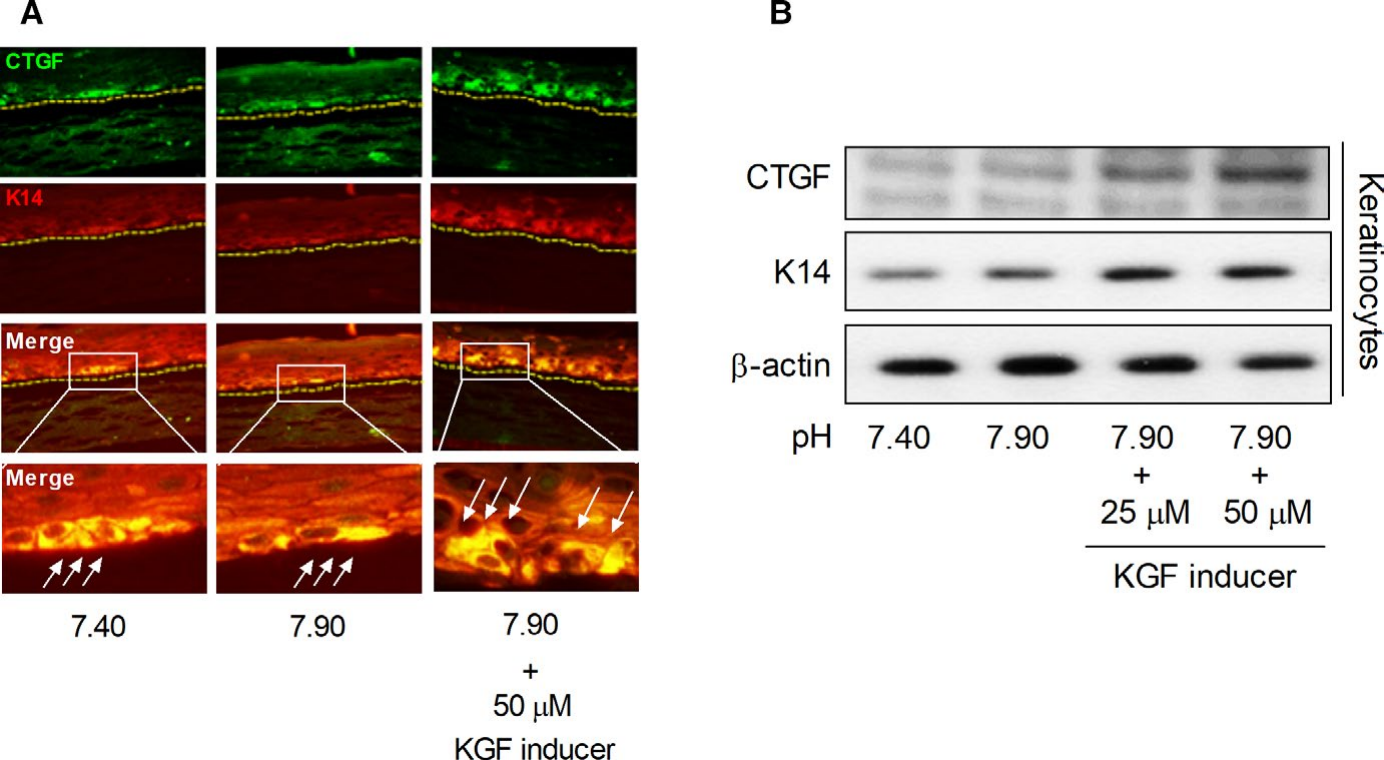
Effects of basic pH and KGF inducer on the CTGF and K14 levels. The level of CTGF and K14 expressions in the 3HSE were measured by IHC (A) and Western blotting (B)

To confirm the effect of basic pH and KGF inducer on skin proliferation, we determined the CTGF and K14 levels. The expression of CTGF and K14 slightly decreased under basic pH, whereas the expression of CTGF and K14 increased under basic pH and treatment with 50 μmol/L KGF inducer (Figure 6).

## 4 DISCUSSION

In this study, we observed enhanced Nrf2 activity and related regulation of antioxidant response enzymes induced by extracellular basic pH in the 3HSE. Furthermore, we observed that extracellular basic pH decreased KGF expression in the 3HSE. These changes are important effectors of a healthy skin.

To evaluate the effect of basic pH on Nrf2 and its regulation of response enzymes in the 3HSE, we evaluated GSH, SOD, CAT, NQO1, and HO‐1 expressions. Previously, we reported that extracellular basic pH increases intracellular ROS production, and this effect has been attributed to its effects on skin ageing and wound recovery.^6^ Generally, one mechanism by which cells may combat oxidative insult is via increased transcription of genes containing the ARE.^20^ ARE is a cis‐acting enhancer sequence that regulates several cytoprotective genes via the transcription factor Nrf2.^20^ ARE‐regulated genes include HO‐1, NQO1 and glutathione‐S‐transferases, as well as those encoding glutathione‐synthesizing enzymes such as glutamate‐cysteine ligase catalytic subunit, glutamate‐cysteine ligase modifier subunit, SOD and CAT.^20^, ^21^ In this study, the 3HSE exposed to basic pH exhibited significantly elevated DNA‐binding activity of Nrf2 and its related response enzyme (ARE‐regulated proteins). It has been shown that skin pH is sensitive to p‐p38 mitogen‐activated protein kinase (MAPK) expression and that it slightly changes in response to pERK and pJNK expression.^6^ The MAPK signal is considered to be affected by the MAPK‐pERK, pJNK and pp38 signalling pathways, and it directly affects Nrf2 activation.^22^, ^23^, ^24^ Further research using MAPK inhibitors is needed to obtain clearer results in this regard.

**FIGURE 7:**
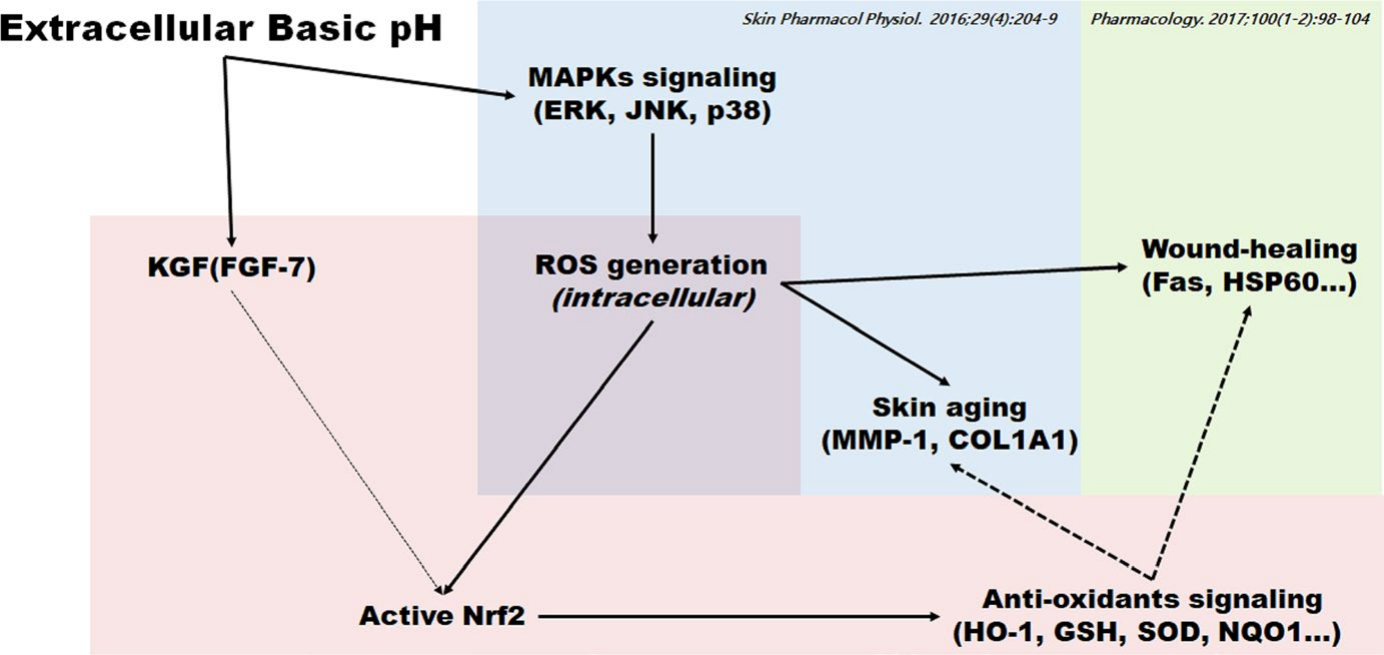
Schematic representation of the mechanisms affected by extracellular basic pH

Keratinocytes form the outermost layer of the skin exposed to the environment.^25^ To ensure skin's resistance to external factors, normal cells in the deeper layers of the epidermis must exhibit a high rate of proliferation, without disturbing the structure and functions.^25^ It is considered that the high resistance of the skin to external factors and its quick response to damages are related to the presence of specific receptors for KGF on the surface of keratinocytes, produced by mesenchymal cells.^25^, ^26^ KGF is a small protein that binds to specific receptors on the keratinocyte cell membrane, and it is a signal for cell proliferation and for new epidermal layer formation in the injured site.^11^, ^26^ The precise mechanism of KGF action is not completely elucidated, but a direct effect of KGF on the increase in Nrf2 activity has been proposed.^11^ It has been reported that the expression of various key players involved in wound healing was significantly reduced in the early phases of wound healing in Nrf2 knockout mouse, and the late phase of wound repair was characterized by prolonged inflammation.^11^ The normal healing rate appears to be at least partially related to the up‐regulation of the related transcription factor Nrf3, which was also identified as a target of KGF and was co‐expressed with Nrf2 in healing skin wounds.^11^ Studies have highlighted the importance of pH control for a healthy skin. In addition, Nrf2 has been confirmed to be an important factor associated with healthy skin, diseases, and ageing.^27^ Therefore, in this study, we measured the changes in KGF expression at different pH levels, and this has not been reported so far. Thus, we evaluated the DNA‐binding activity of Nrf2 after treatment with KGF inducer in an extracellular basic pH model. Our results showed that the treatment with KGF inducer along with extracellular basic pH significantly increased Nrf2‐DNA‐binding activity compared with extracellular basic pH treatment. Moreover, KGF and extracellular basic pH treatment significantly increased the GSH, HO‐1, SOD and CAT levels compared with the extracellular basic pH treatment. However, there was no considerable difference between the KGF inducer‐treated group and the untreated group at pH 7.70; this indicated that the DNA‐binding activity of Nrf2 and its regulation of antioxidative defence enzymes are sensitive to oxidative stresses such as ROS production, than KGF level. These results suggest that basic pH conditions increased Nrf2 activity and antioxidative defence system activity, but decreased KGF expression. Moreover, the CTGF and K14 levels were determined to confirm the changes in skin cell connective tissue growth and skin proliferation by pH regulation. We found that they were slightly reduced (Figure 6). However, KGF plays an important role in skin connective tissue growth and skin cell proliferation because they were increased by treatment with KGF. Additional studies and similar clinical models are required to further elucidate the mechanism underlying this phenomenon. Based on previous papers and the results of this paper, given that the antioxidant effect increases when the KGF inducer is processed, pH control and KGF inducer can play an important role in skin ageing or wound healing in the future (Figure 7).^6^, ^7^ Importantly, the inhibition of wound healing through pH imbalance and KGF regulation suggests that the development of pH‐regulating or pH‐maintaining materials may provide effective therapeutic strategies for maintaining a healthy skin.

Future studies should perform metabolic profiling under different pH conditions. The analysis of NADPH activity is an important part of oxidative stress studies; we just briefly checked this in HaCaT cell line, and therefore, more detailed research is needed in the future (Figure S3). Moreover, we further confirmed KGF inhibition or Nrf2 inhibition (ML385) (Figures S4 and S5) in HaCaT cell line, but this was not confirmed using the 3HSE system. Additionally, animal models in which pH can be reduced or increased are necessary. We believe that our experimental methods could be used for screening skin health‐related drugs; they can also be used in modelling studies of skin diseases by pH control in the future.

## CONFLICT OF INTEREST

The authors declare no conflicts of interest.

## AUTHOR CONTRIBUTIONS

**Gunhyuk Park:** Conceptualization (lead); data curation (lead); funding acquisition (lead); investigation (lead); methodology (lead); project administration (lead); writing‐original draft (lead). **Byeong Cheol Moon:** Data curation (supporting); writing‐original draft (supporting). **Dal‐Seok Oh:** Methodology (supporting); validation (supporting); writing‐original draft (equal). **Yong‐Ung Kim:** Conceptualization (equal); investigation (supporting); writing‐original draft (equal); writing‐review & editing (equal). **Moon‐Ki Park:** Conceptualization (equal); writing‐original draft (equal); writing‐review & editing (equal).

## Supporting information

Fig S1

Fig S2

Fig S3

Fig S4

Fig S5

Table S1‐S6

## Data Availability

The data that support the findings of this study are available from the corresponding author upon reasonable request.
