# Supplementary figures and images for: Enhanced Nrf2 up‐regulation by extracellular basic pH in a human skin equivalent system
Source: J Cell Mol Med. 2021 Mar 16;25(7):3646–53. doi: 10.1111/jcmm.16472 (PMC8034446; doi:10.1111/jcmm.16472)

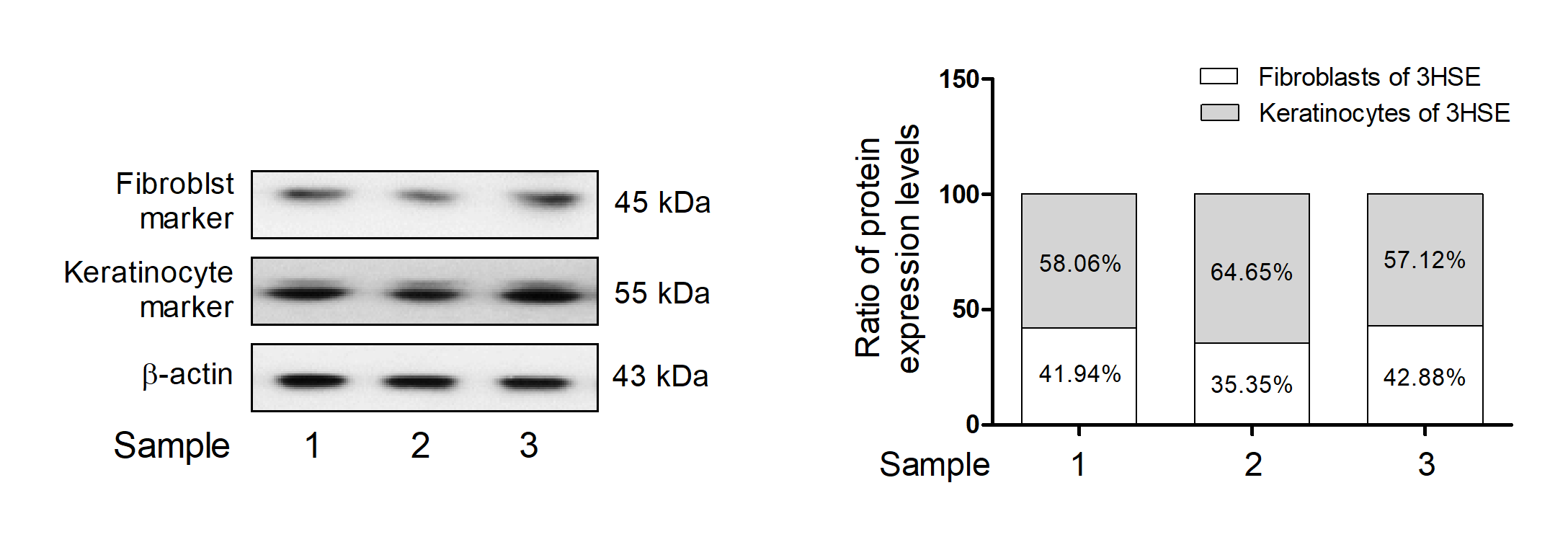

Supplement: Supplementary file 1 — Fig S1 [file JCMM-25-3646-s006.tif]

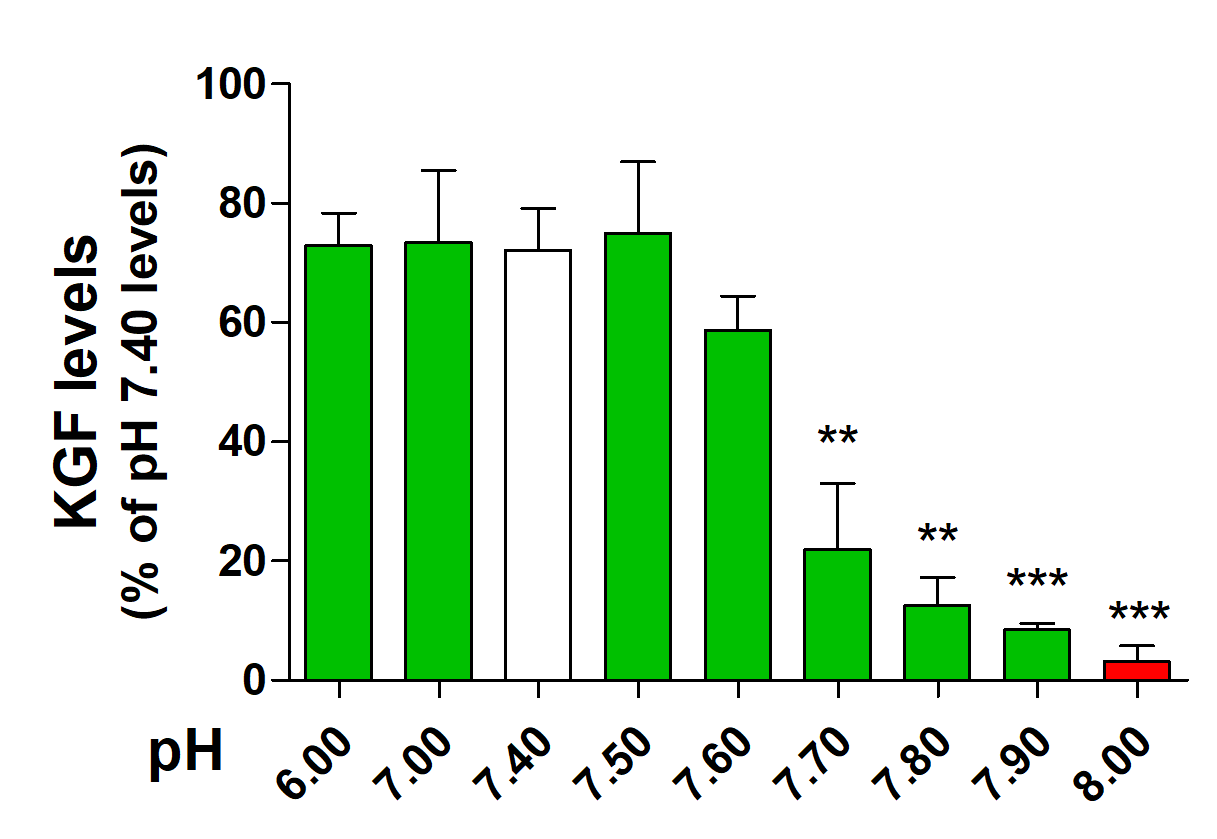

Supplement: Supplementary file 2 — Fig S2 [file JCMM-25-3646-s005.tif]

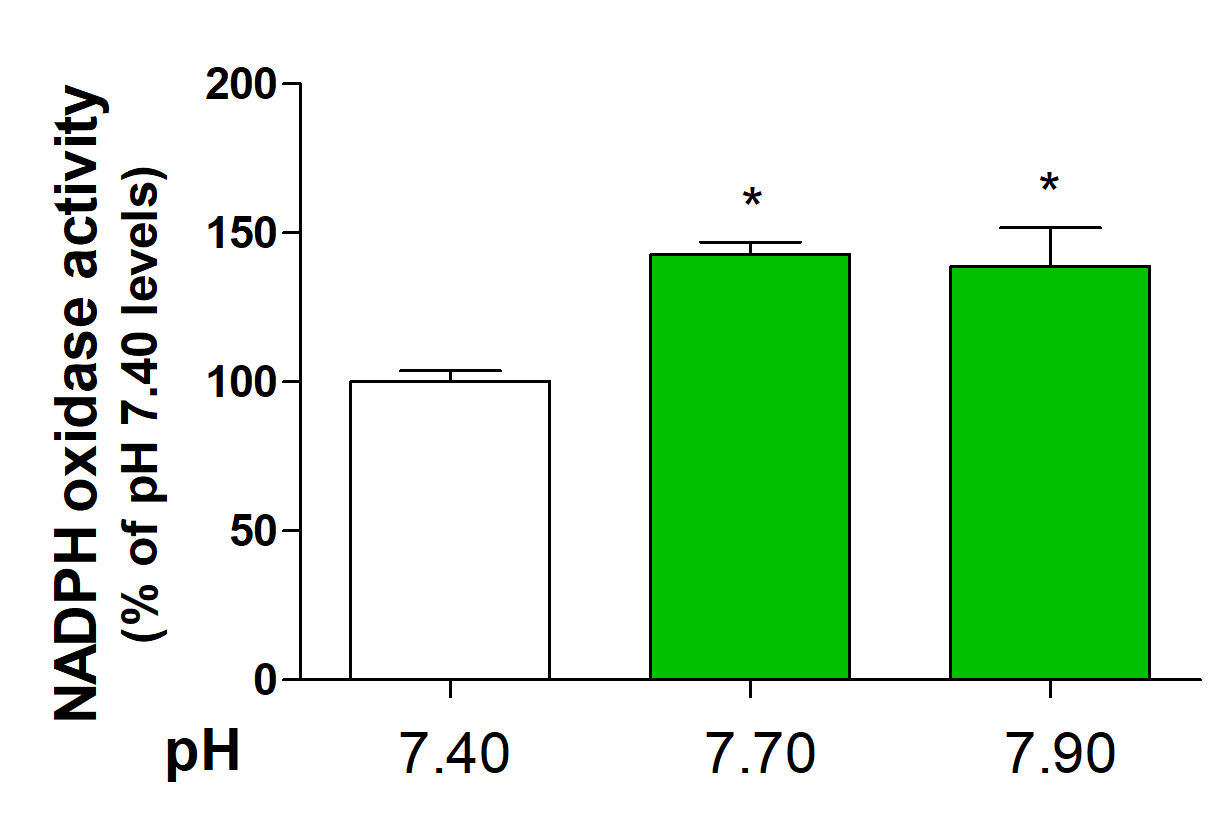

Supplement: Supplementary file 3 — Fig S3 [file JCMM-25-3646-s001.tif]

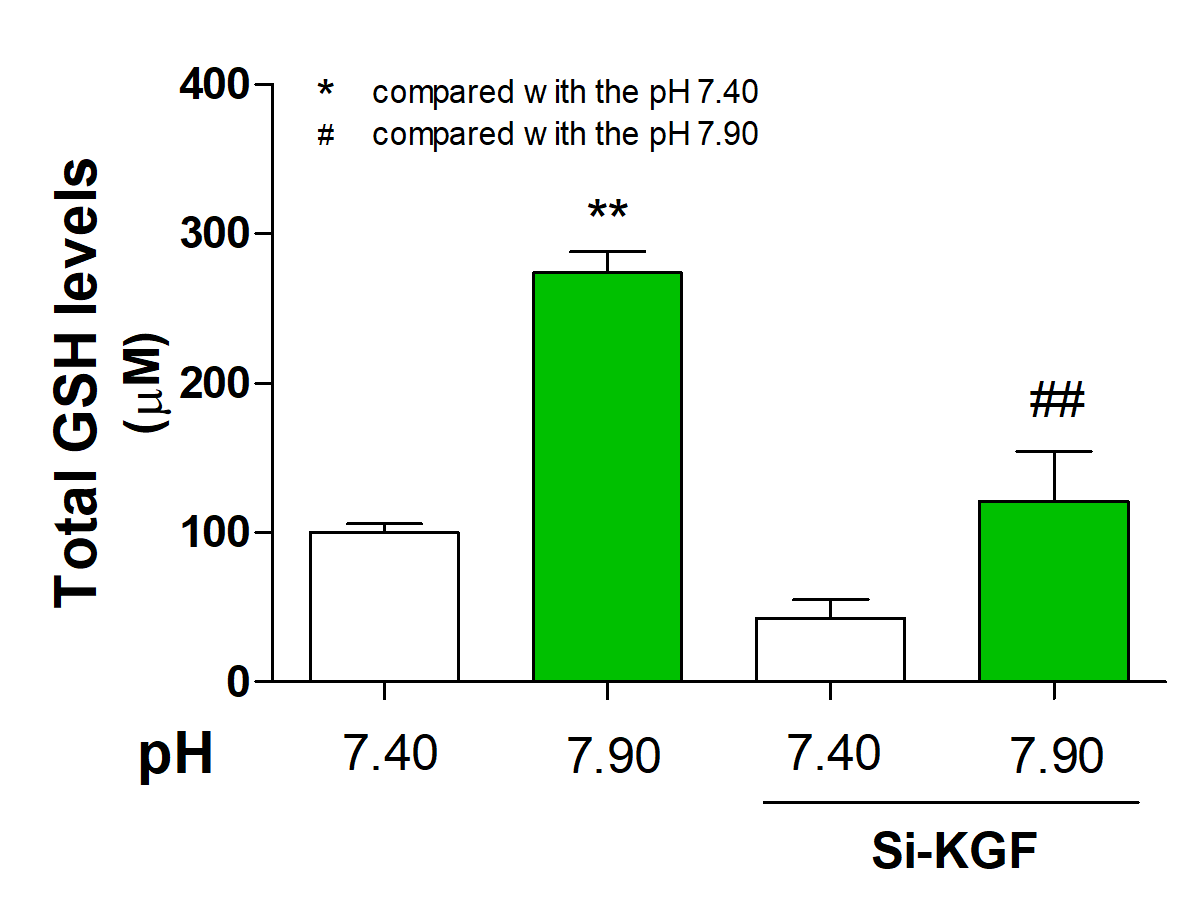

Supplement: Supplementary file 4 — Fig S4 [file JCMM-25-3646-s004.tif]

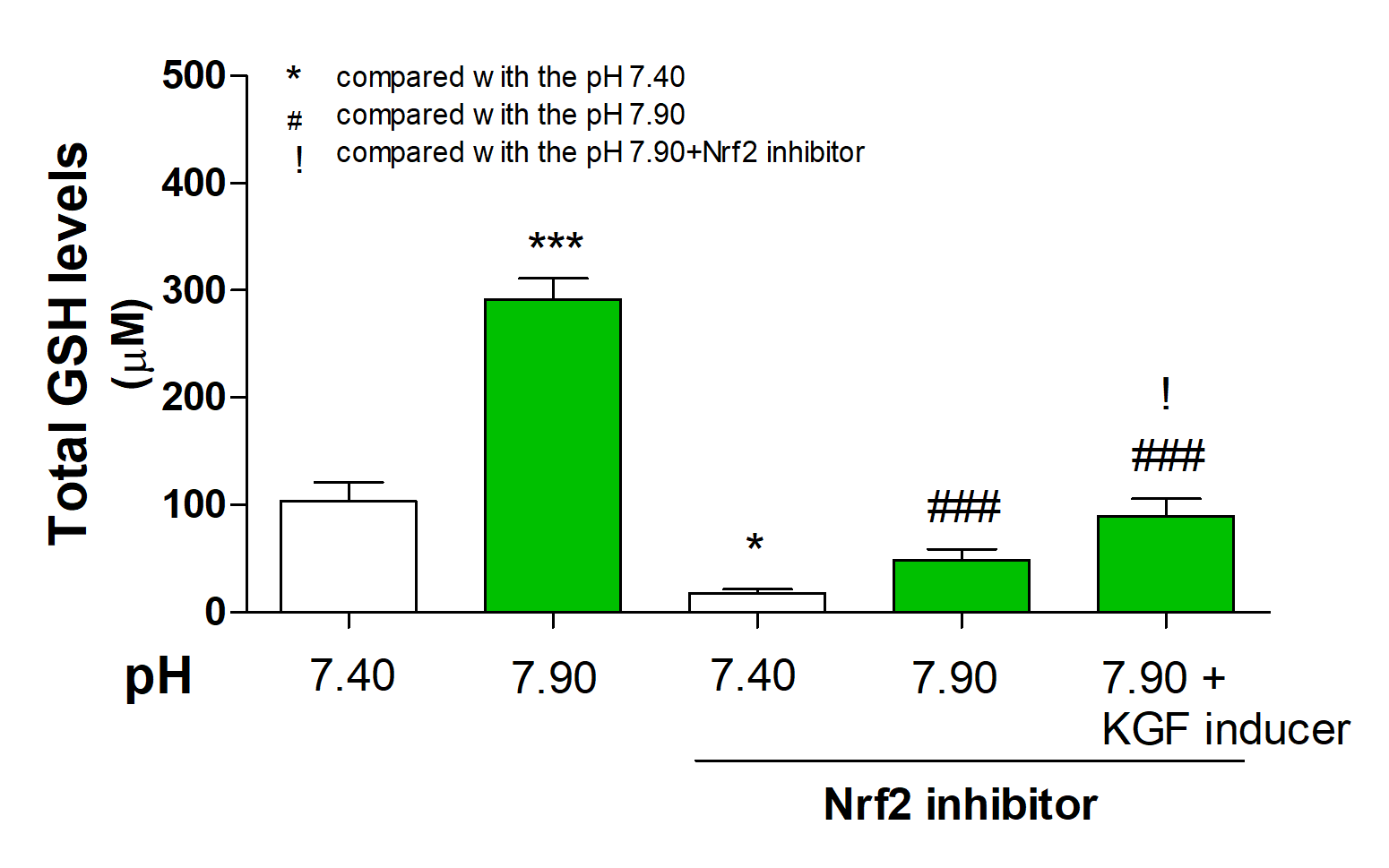

Supplement: Supplementary file 5 — Fig S5 [file JCMM-25-3646-s002.tif]
