# Supplementary material for: Enhanced Nrf2 up‐regulation by extracellular basic pH in a human skin equivalent system
Source: J Cell Mol Med. 2021 Mar 16;25(7):3646–53. doi: 10.1111/jcmm.16472 (PMC8034446; doi:10.1111/jcmm.16472)
Supplement: Supplementary file 6 — Table S1‐S6 [file JCMM-25-3646-s003.docx]

| **pH** | **7.00** | **7.40** | **7.70** | **7.90** |
| --- | --- | --- | --- | --- |
| **Nrf2 DNA binding activity (% of pH 7.40 levels)** | 94.95±7.66 | 100±6.94 | 197.35±9.43 | 235.45±4.90 |

**Supplementary Table 1**

**Supplementary Table 2**

|  | **pH** | **7.00** | **7.40** | **7.70** | **7.80** | **7.90** |
| --- | --- | --- | --- | --- | --- | --- |
| **(A)** | **Total GSH levels (mM)** | 0.87±0.34 | 1.52±0.21 | 3.11±0.55** | 3.99±0.38*** | 4.27±0.23*** |
| **(B)** | **SOD levels (% of pH 7.4 levels)** | 102.02±0.67 | 100±0.35 | 178.56±9.69*** | 173.17±8.92*** | 190.67±14.3*** |
| **(C)** | **CAT activity (mM/mL)** | 3.50±0.22 | 3.83±0.23 | 4.92±0.13** | 4.89±0.10** | 4.82±0.11** |
| **(D)** | **NQO1 levels (% of pH 7.40 levels)** | 64.19±17.43 | 100±28.12 | 270.69±41.70** |  | 291.55±27.31** |
| **(E)** | **HO-1 levels (% of pH 7.40 levels)** | 100.97±21.81 | 100±21.74 | 174.58±16.46* |  | 191.56±4.87* |

**Supplementary Table 3**

|  | **pH** | **6.00** | **7.00** | **7.40** | **7.50** | **7.60** | **7.70** | **7.80** | **7.90** | **8.00** |
| --- | --- | --- | --- | --- | --- | --- | --- | --- | --- | --- |
| **(A)** | **Cytotoxicity (% of pH 7.40 levels)** | 101.31±2.49 | 105.63±6.75 | 100.93±3.38 | 98.23±5.23 | 96.88±2.54 | 97.47±3.69 | 98.15±4.12 | 99.86±7.16 | 162.84±5.36*** |
|  |  |  |  |  |  |  |  |  |  |  |
|  | **pH** | **7.40** | **7.50** | **7.60** | **7.70** |  |  |  |  |  |
| **(B)** | **KGF levels (% of pH 7.40 levels)** | 100.00±5.57 | 89.30±5.39 | 80.50±4.04 | 66.87±3.24* |  |  |  |  |  |

**Supplementary Table 4**

| **pH** | **7.40** | **7.70** | **7.90** | **7.40** | **7.70** | **7.90** | **7.40** | **7.70** | **7.90** |
| --- | --- | --- | --- | --- | --- | --- | --- | --- | --- |
| **KGF inducer** |  |  |  | **25 μM** | **25 μM** | **25 μM** | **50 μM** | **50 μM** | **50 μM** |
| **Nrf2 DNA-binding activity (% of pH 7.40 levels)** | 100  ±10.21 | 245.04  ±29.51^***^ | 409.25  ±5.01^***^ | 263.91  ±5.68^***^ | 321.43  ±7.69^##, !!!^ | 429.17  ±15.69^###, @@@^ | 323.31  ±10.53^***, ##^ | 396.77  ±34.36^!!!, $$^ | 540.23  ±8.19^@@@, $$$^ |

**Supplementary Table 5**

| **pH** | **7.40** | **7.40** | **7.70** | **7.90** | **7.40** | **7.40** | **7.70** | **7.90** |
| --- | --- | --- | --- | --- | --- | --- | --- | --- |
| **KGF inducer** |  | **25 μM** | **25 μM** | **25 μM** |  | **50 μM** | **50 μM** | **50 μM** |
| **HO-1 levels (% of pH 7.40 levels)** | 100.00  ±2.54 | 197.81  ±14.68^***^ | 240.21  ±14.23^***^ | 324.24  ±1.51^***^ | 100.00  ±7.68 | 205.29  ±8.07^***^ | 293.37  ±8.14^***^ | 340.56  ±8.71^***^ |
| **Total GSH levels (mM)** | 1.17  ±0.08 | 5.42  ±0.29^***^ | 6.20  ±0.37^***^ | 9.42  ±0.67^***^ | 0.94  ±0.23 | 7.69  ±0.70^***^ | 10.77  ±0.64^***^ | 15.06  ±0.27^***^ |
